# Beyond words: developing a scale to measure the embodied professional literacy of K-12 physical education teachers

**DOI:** 10.3389/fpsyg.2026.1789350

**Published:** 2026-05-15

**Authors:** Zhi Li, Jingdong Jia, Zijing Jiang, Kai Ding, Chenlin Li, Xiang Zou

**Affiliations:** 1 School of Physical Education, XI'an University, Xi'an, China; 2 School of Physical Education, Shaanxi Normal University, Xi'an, China

**Keywords:** Embodied professional literacy, K-12 education, physical education teachers, psychometrics, scale development

## Abstract

**Background:**

In physical education (PE), the teacher's body serves as the primary instrument of instruction. However, existing evaluations often fail to capture this context-specific “embodied” nature, typically reducing professional competency to either abstract cognitive beliefs or generic physical fitness.

**Objective:**

To address this epistemological gap, this study conceptualizes and psychometrically validates the Physical Education Teachers' Embodied Professional Literacy Scale (PE-TEPLS), innovatively operationalizing the “pedagogical body” in the K-12 context.

**Methods:**

A rigorous mixed-method design was employed, combining Delphi consultation (*N* = 20), the Analytic Hierarchy Process (AHP), and a cross-sectional survey of 1,665 PE teachers in China. Data underwent Exploratory and Confirmatory Factor Analysis (EFA/CFA). CFA corroborated this correlated model, and Multi-Group CFA confirmed scalar invariance across gender. Reliability and discriminant validity were robustly established.

**Results:**

Psychometric analyses confirmed a robust 45-item structure across five dimensions: embodied moral cultivation, motor ability basis, situational interaction, teaching transformation, and lifelong development. CFA corroborated this correlated model, and Multi-Group CFA confirmed scalar invariance across gender. Reliability and discriminant validity were robustly established.

**Conclusion:**

The PE-TEPLS provides a psychometrically robust and equitable instrument for assessing PE teachers. By theoretically legitimizing “embodied action” and prioritizing the “pedagogical body” over the “athletic body,” this study offers a new paradigm for evaluating teacher professionalism beyond verbal and cognitive metrics.

## 1 Introduction

Physical literacy (PL) has gained worldwide acknowledgment as a fundamental aspect for sustaining lifelong participation in physical activities and plays a vital role in overall health (Hastie and Wallhead, 2015). Consequently, fostering PL has become a central goal of Quality Physical Education (QPE), positioning physical education (PE) instructors as crucial facilitators and implementers within this global framework (UNESCO, 2015). However, the role of the body for a PE teacher transcends this foundational, individual-level concept of wellness. In the pedagogical context, the teacher's body serves not merely as a site of personal health but as the primary instrument of instruction (Silverman and Mercier, 2015). Unlike educators in other disciplines who primarily rely on verbal or symbolic communication, PE teachers must skillfully employ their physical presence to demonstrate complex motor skills, ensure student safety through proactive positioning and intervention, and manage classroom dynamics through nuanced non-verbal cues (Riley and Proctor, 2023). This performative and relational use of the body transforms a teacher's physical competence from a personal attribute into a form of essential professional capital.

Despite the theoretical consensus on the importance of embodiment (Whitehead, 2010), a significant gap remains in its operationalization. Conventional assessments often focus on the individual's capacity for movement and self-expression (Pavlova, 2023), or are predominantly anchored in cognitivist paradigms (Di Fabio and Palazzeschi, 2008). Prevailing tools, such as the Perceived Physical Literacy Instrument (PPLI) (Choi et al., 2018) and various teacher efficacy scales, typically operationalize literacy through self-reported “confidence” or “beliefs” (Sheldon et al., 2020). This approach often neglects the “performative” dimension of PE teaching, reducing the lived, situated experience of the body to abstract cognitive judgments. Compounding this measurement limitation is a contextual deficit. Most existing scales are designed for the general population, fundamentally ignoring the distinct pedagogical ecology of the K-12 gymnasium-such as dynamic safety management and instructional demonstrations. This gap limits the validity of assessments for PE teachers (Lyu, 2021), highlighting the urgent need for an instrument rooted in the practical realities of embodied teaching practice.

To effectively operationalize this construct, we synthesized theoretical frameworks and expert insights to propose five core dimensions of Embodied Professional Literacy (EPL). These include: (1) Embodied Moral Cultivation, reflecting ethical conduct enacted through physical presence (Gerdin et al., 2021; Noddings, 1984); (2) Motor Ability Basis, covering foundational movement competence required for accurate skill demonstration (Shearer et al., 2018); (3) Teaching Transformation, the ability to translate personal skills into student learning via cognitive conversion and differentiated instruction (Anderson, 1982; van Munster et al., 2019); (4) Situational Interaction, involving dynamic risk prediction and spatial awareness (Vert et al., 2021); and (5) Lifelong Development, focusing on occupational health and role-modeling a healthy lifestyle (Markelj et al., 2024; Hamilton et al., 2021). This multidimensional framework moves beyond individual physical capacity to capture the relational and pedagogical enactment of the teacher's body (Roetert et al., 2018).

To bridge the gap between theoretical embodiment and empirical assessment, the primary aim of this study is to develop and psychometrically validate the Physical Education Teacher Embodied Professional Literacy Scale (PE-TEPLS) for K-12 teachers in China. To ensure methodological rigor, this study employs a mixed-method design combining Delphi consultation and the Analytic Hierarchy Process (AHP) for instrument development, followed by Principal Axis Factoring (PAF) with Promax rotation and Multi-Group Confirmatory Factor Analysis (MGCFA). By operationalizing the “pedagogical body,” the PE-TEPLS seeks to provide a valid, reliable, and equitable tool that refocuses assessment on how teachers skillfully use their physical presence to teach, protect, and inspire students.

## 2 Methods

### 2.1 Study design and ethical considerations

This study adopted a multi-phase scale development and validation design. The research process was structured to transition from theoretical construction and item generation (deductive-inductive approach) to quantitative psychometric validation (cross-sectional survey; MacKenzie et al., 2011). This design ensures that the developed instrument is both theoretically grounded and empirically robust across different samples (Stefana et al., 2025). All participants provided informed consent prior to data collection. They were explicitly informed of the study's purpose, the voluntary nature of their participation, and their right to withdraw at any time. To ensure confidentiality, all data were anonymized, and personal identifiers were removed before analysis.

### 2.2 Participants and sampling strategy

Participants were strategically recruited across four phases to align with specific research objectives, balancing theoretical depth with broad generalizability.

#### 2.2.1 Phases 1–3

Scale development and pilot in Phase 1 (Theoretical Construction & Item Generation), a purposive sample of 32 experts and frontline teachers was consulted to ensure the theoretical constructs were effectively operationalized into observable teaching behaviors (Sankofa, 2021). This sample was stratified to include university professors (for theoretical insight) and K-12 teachers from diverse school settings and career stages (for practical perspective). For Phase 2 (Delphi Consultation), the panel was structurally optimized to 20 experts. While the Phase 1 sample prioritized data saturation, the Delphi phase focused on expert consensus efficiency. Therefore, we streamlined the panel by reducing redundancy among frontline practitioners while retaining high-authority experts. To enhance the scale's contextual validity and policy relevance, this optimized panel was deliberately expanded beyond academics to include school leaders and education officials, with specific representation from border regions (e.g., Xinjiang, Yunnan) to capture geographically diverse pedagogical challenges. The pilot study (Phase 3) utilized a convenience sample of 128 PE teachers from Shaanxi Province, a size consistent with recommendations for preliminary item analysis (e.g., at least 5–10 participants per item).

#### 2.2.2 Phase 4

For the formal validation, a large-scale cross-sectional survey was administered from May to September 2025 using the Wenjuanxing platform. To ensure a representative sample, we employed a stratified sampling strategy covering 28 provinces in mainland China. The target population was stratified by geographic region (East, Central, West), school level (Primary, Junior High, Senior High), and urban-rural location.

We initially collected 2,047 responses and applied a three-stage screening protocol to ensure data quality. First, we excluded respondents who failed any of the four embedded attention checks (e.g., agreeing to “I wear a formal suit for every PE class”). Second, we removed responses completed in under 120 seconds, as pilot testing indicated this was insufficient for valid completion. Third, we discarded surveys showing invariant response patterns (“straight-lining”) or logical inconsistencies.

This strict protocol resulted in the exclusion of 382 invalid responses, yielding a final valid sample of 1,665 teachers (Effective Response Rate = 81.3%). This sample size not only meets but exceeds common subject-to-item ratio recommendations for factor analysis (Lingard and Rowlinson, 2006), ensuring robust results. The final sample achieved a balanced geographic representation: East China (~38.5%), Central China (~34.2%), and West China (~27.3%). The demographic composition included 61.9% from urban areas, 20.7% from counties, and 16.8% from rural villages, with a gender ratio of 64.7% male to 35.3% female. Detailed demographic characteristics are presented in Table 1.

**Table 1:** Demographic characteristics of the participants (Total *N* = 1,665).

Characteristic	Category	Total Sample (*N* = 1,665) *n* (%)	Subsample A (EFA; *n* = 793) *n* (%)	Subsample B (CFA; *n* = 872) *n* (%)
Gender	Male	1,076 (64.62)	513 (64.69)	563 (64.56)
	Female	589 (35.38)	280 (35.31)	309 (35.44)
Age (years)	≤ 25	232 (13.93)	109 (13.75)	123 (14.11)
	26–35	1,177 (70.69)	566 (71.37)	611 (70.07)
	36–45	229 (13.75)	106 (13.37)	123 (14.11)
	≥46	27 (1.62)	12 (1.51)	15 (1.72)
Geographic region	East China	641 (38.50)	305 (38.46)	336 (38.53)
	Central China	569 (34.17)	271 (34.17)	298 (34.17)
	West China	455 (27.33)	217 (27.37)	238 (27.30)
Professional title	Third-grade	232 (13.93)	109 (13.75)	123 (14.11)
	Second-grade	960 (57.66)	462 (58.26)	498 (57.11)
	First-grade	414 (24.86)	195 (24.59)	219 (25.11)
	Senior	59 (3.54)	27 (3.40)	32 (3.67)
School level	Primary school	1,044 (62.70)	503 (63.43)	541 (62.04)
	Junior high school	332 (19.94)	157 (19.80)	175 (20.07)
	Senior high/vocational	289 (17.36)	133 (16.77)	156 (17.89)
School location	Urban (City)	1,006 (60.42)	491 (61.92)	515 (59.06)
	County	348 (20.90)	164 (20.68)	184 (21.10)
	Rural (Township)	273 (16.40)	133 (16.77)	140 (16.06)
	Border/Ethnic Regions	38 (2.28)	5 (0.63)	33 (3.78)
Teaching experience	≤ 5 years	716 (43.00)	341 (43.00)	375 (43.00)
	6–10 years	686 (41.20)	328 (41.36)	358 (41.06)
	11–15 years	167 (10.03)	79 (9.96)	88 (10.09)
	16–20 years	50 (3.00)	23 (2.90)	27 (3.10)
	>20 years	46 (2.76)	22 (2.77)	24 (2.75)
Weekly workload	≤ 10 sessions	239 (14.35)	108 (13.62)	131 (15.02)
	11–15 sessions	491 (29.49)	229 (28.88)	262 (30.05)
	16–20 sessions	786 (47.21)	384 (48.42)	402 (46.10)
	≥21 sessions	149 (8.95)	72 (9.08)	77 (8.83)
Self-rated health	Very good	328 (19.70)	153 (19.29)	175 (20.07)
	Good	823 (49.43)	395 (49.81)	428 (49.08)
	Fair	495 (29.73)	233 (29.38)	262 (30.05)
	Poor	19 (1.14)	12 (1.51)	7 (0.80)
Exercise frequency	≥3 times/week	501 (30.09)	239 (30.14)	262 (30.05)
	1–2 times/week	737 (44.26)	353 (44.51)	384 (44.04)
	1–3 times/month	301 (18.08)	144 (18.16)	157 (18.00)
	Rarely/Never	126 (7.57)	57 (7.19)	69 (7.91)

### 2.3 Measures and procedure

#### 2.3.1 Measures

The primary measure developed and validated in this study was the Physical Education Teachers' Embodied Professional Literacy Scale (PE-TEPLS). In its final form, it comprises 45 items across 10 factors and 5 dimensions. All items were measured using a 5-point Likert scale (Willits et al., 2016) ranging from 1 (Strongly Disagree) to 5 (Strongly Agree). To establish criterion validity, the Emotional Awareness Scale (EAS) was administered concurrently, as theoretical frameworks posit a strong association between embodied cognition and emotional regulation in teaching contexts (Bu and Huili, 2020).

#### 2.3.2 Procedure

**Figure 1:**
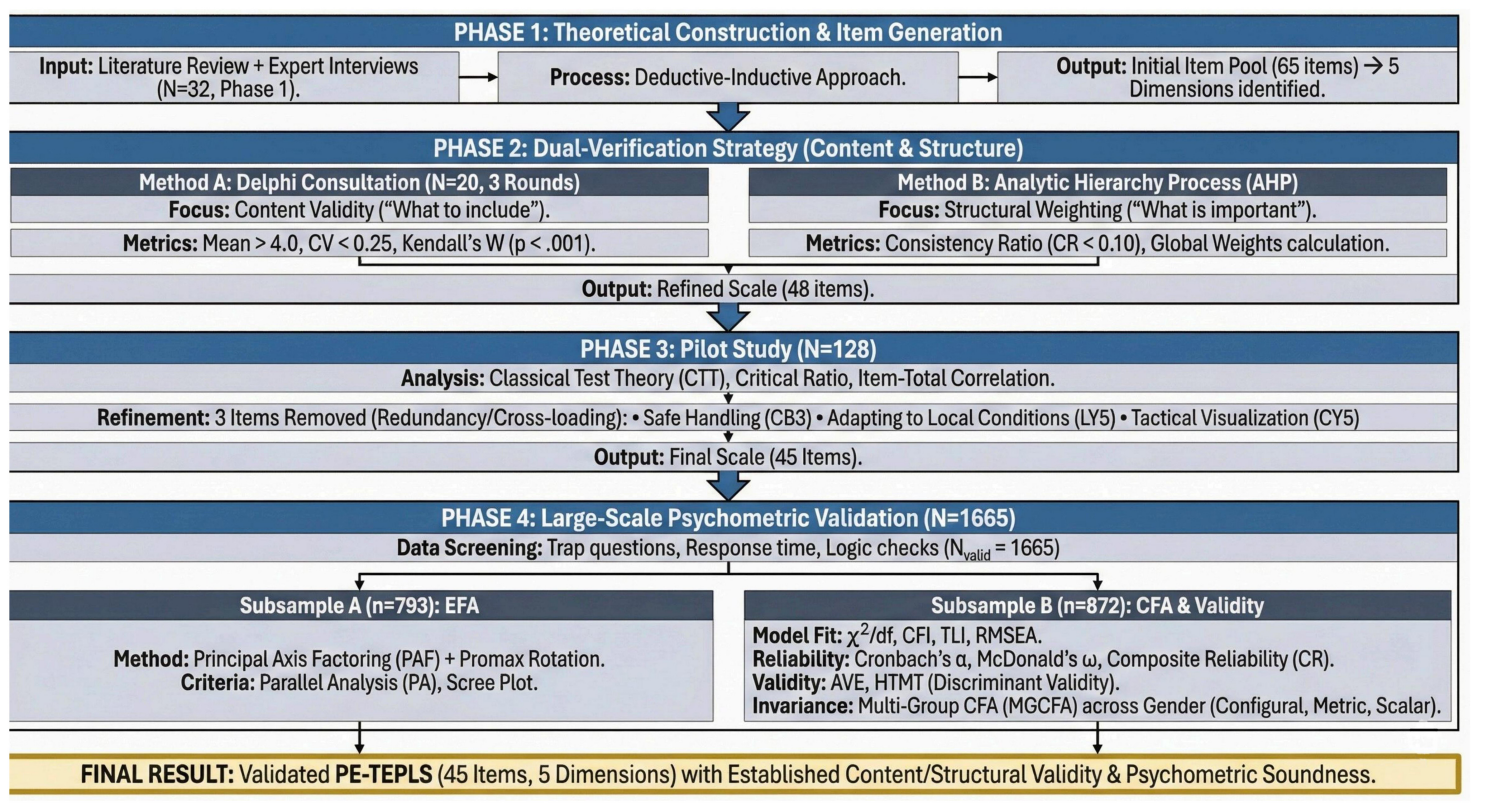
Methodological framework for developing and validating the PE-TEPLS.

The development and validation of the PE-TEPLS followed a rigorous multi-phase procedure, involving theoretical construction, expert consultation, and psychometric testing. The complete methodological flowchart is presented in Figure 1. The process unfolded sequentially across four phases:

#### 2.3.3 Phase 1 (Item Generation)

First, a comprehensive literature review was conducted to identify theoretical dimensions of embodied cognition and professional literacy. Subsequently, unstructured interviews

and expert consultations were held with university professors and frontline PE teachers to capture the nuances of teaching practices. Insights gained from these discussions were used to refine the theoretical constructs and generate the initial 65-item pool, ensuring the items were ecologically valid and contextually relevant.

#### 2.3.4 Phase 2 (Content validity and structural weighting)

A three-round Delphi consultation was conducted with a panel of 20 experts to screen the initial item pool. Items were rated on a 5-point scale for relevance and clarity, with strict retention criteria applied (Mean >4.0, CV < 0.25). Crucially, to ensure both comprehensive coverage and structural precision, this phase adopted a dual-verification strategy integrating Delphi and the Analytic Hierarchy Process (AHP). While the Delphi method established content validity (“what to include”) by qualitatively refining items, it often suffers from a “ceiling effect” where experts rate all retained items as highly important. To resolve this, AHP was subsequently applied to determine the structural hierarchy (“what is important”). By mandating pairwise comparisons, AHP quantifies the relative weight of each dimension. Furthermore, the reliability of expert consensus was mathematically verified via the AHP Consistency Ratio (CR), with a threshold of CR < 0.10 ensuring logical consistency in the experts‘ judgments (Saaty, 1980). Expert insights were also integrated to refine item wording and structure (Table 2).

**Table 2:** Composition and qualifications of the expert panel (Delphi phase, *N* = 20).

Category	Role/position	Sample (*n*)	Percentage (%)	Rationale for inclusion
Academic experts	University professors (sports pedagogy/psychometrics)	8	40.00%	Theoretical depth and methodological rigor
Frontline practitioners	Senior PE teachers (urban/county schools)	5	25.00%	Practical instructional experience
	Border region teachers (e.g., Xinjiang, Yunnan)	3	15.00%	Cultural inclusivity and regional diversity
Educational admin.	School principals (primary/secondary)	2	10.00%	Management and implementation perspective
	Education bureau officials (local/provincial)	2	10.00%	Policy alignment and curriculum standards
Total		20	100.00%	

Quantitative validity metrics: the expert panel demonstrated high authority with coefficient values (Cr) ranging from 0.749 to 0.764 (Benchmark >0.70).

Consensus was successfully reached in Round 3, with a statistically significant Kendall's coefficient of concordance: W = 0.262 (p < 0.001), indicating robust agreement among experts from diverse backgrounds.

#### 2.3.5 Phase 3 (Pilot Testing)

The 48-item draft scale was administered online to a pilot sample (*N* = 128). Item analysis followed established psychometric guidelines: the Critical Ratio (CR) method was used to compare extreme groups (top 27% vs. bottom 27%; Zhong et al., 2023), with a CR >3.0 indicating adequate discrimination; concurrently, item-total correlations were computed, retaining items with correlations >0.40. This led to the removal of three items. To evaluate temporal stability, a test-retest procedure was implemented. A random subsample of 50 participants from the pilot phase completed the scale again after a two-week interval.

#### 2.3.6 Phase 4 (Psychometric)

The finalized 45-item scale was distributed nationally (*N* = 1,665). To ensure confidentiality, all data were anonymized, and personal identifiers were removed before analysis. Data cleaning involved attention checks and minimum response time filters (Gummer et al., 2018). The valid dataset was randomly split into two subsamples for separate analyses (Shaw et al., 2015): subsample A (*n* = 793) for Exploratory Factor Analysis (EFA) using Principal Axis Factoring with Promax rotation (Griffiths et al., 2006), and Subsample B (*n* = 872) for Confirmatory Factor Analysis (CFA), reliability assessment, and tests of convergent/discriminant validity (Rönkkö and Cho, 2020).

### 2.4 Statistical analysis

All analyses were conducted using SPSS 27.0, AMOS 26.0, and SmartPLS 4.

For the pilot study (*N* = 128) and exploratory validation (Subsample A, *n* = 793), Classical Test Theory (CTT) was applied using SPSS. Item analysis retained items based on Critical Ratio (CR >3.00) and item-total correlations (>0.40). Internal consistency was assessed using Cronbach's α and McDonald's Omega (ω), with values >0.70 considered acceptable.

Exploratory Factor Analysis (EFA) was performed on Subsample A (KMO >0.80, Bartlett's *p* < 0.001). Principal Axis Factoring (PAF) with Promax rotation was employed to extract latent constructs. The number of factors was determined by Parallel Analysis (PA), eigenvalues >1.0, and scree plot inspection.

For confirmatory validation (Subsample B, *n* = 872), Confirmatory Factor Analysis (CFA) was conducted using AMOS 26.0 to rigorously test the hypothesized Second-Order Hierarchical Model. In this specification, the 45 items load onto 10 first-order factors, which subsequently load onto five underlying second-order dimensions (e.g., Embodied Moral Cultivation, Motor Ability Basis). Model fit was evaluated via the Chi-square/df ratio (χ^2^/df < 3.0), CFI/TLI (>0.90), and RMSEA (< 0.08). Convergent validity was assessed based on standardized factor loadings (>0.50), Average Variance Extracted (AVE >0.50), and Composite Reliability (CR >0.70). Discriminant validity was rigorously examined using both the Fornell–Larcker criterion and the Heterotrait–Monotrait ratio (HTMT < 0.85). Finally, measurement invariance across gender was tested using Multi-Group CFA (MGCFA), and Common Method Bias (CMB) was diagnosed using the Common Latent Factor (CLF) approach.

## 3 Results

### 3.1 Study 1: theoretical construction and initial item generation

The foundational theoretical architecture was deductively constructed, subsequently enriched by the collective insights of a multidisciplinary expert cohort. This methodological syncretism, integrating extant scholarly discourse on embodied cognition with the practical heuristics gleaned from 32 frontline pedagogical practitioners, facilitated the precise operationalization of Embodied Professional Literacy (EPL) into empirically tractable dimensions. Prioritizing the establishment of content validity and pedagogical salience over prescriptive procedural codification, this initial phase culminated in the crystallization of five cardinal dimensions that underpin the theoretical fabric of EPL: (1) Embodied Moral Cultivation, (2) Motor Ability Basis, (3) Situational Interaction, (4) Teaching Transformation, and (5) Lifelong Development. This quintuple-dimensional schema, meticulously delineated in Table 1, conferred the structural imprimatur for the subsequent proliferation of assessment items.

A discerning review of the emergent item pool evinced a discernible variance in conceptual granularity across its constituent dimensions. The dimension pertaining to Motor Ability Basis manifested a pronounced density of explicit, empirically observable sub-indicators (e.g., precise kinematic demonstration, sustained exertion for guided instructional sequences). Conversely, dimensions such as Lifelong Development were characterized by broader conceptualizations, exhibiting a relative paucity of finely articulated operational descriptors. This differential structural distribution underscored a preponderance of psychomotor-technical referents, juxtaposed against a comparative underrepresentation of developmental and introspective thematic elements within the nascent framework. This preliminary construct, delimited by its identified heterogeneity in item specificity, thus constituted the baseline input for the subsequent Delphi consultation, thereby priming the construct for systematic structural reification and validation (Table 3).

**Table 3:** Core dimensions and supporting expert insights of embodied professional literacy.

Core dimension	Conceptual focus	Illustrative expert insights
1. Embodied moral cultivation	Ethics in action	In uncomfortable moments, such as when a student's clothing rips, I promptly use my own body or jacket to shield them. Taking action to preserve a child's dignity speaks louder than any words could convey.
2. Motor ability basis	Physical competence	A physical education instructor must embody a “walking encyclopedia.” It is essential to possess the endurance to guide the run without faltering, and you must accurately showcase each movement consistently, all while maintaining an appearance of energy.
3. Situational interaction	Safety & environment	I consistently place myself at the perimeter of the classroom during lessons to ensure I catch every moment. When I notice a potential collision, my immediate reaction is to intervene and act as a shield to protect the students.
4. Teaching transformation	Pedagogical body	A firm high-five fosters trust more effectively than spoken compliments. To instruct on a complicated maneuver, I amplify the action or employ a “mirror demonstration” to assist learners in understanding the correct movements.
5. Lifelong development	sustained growth	Maintaining my physical fitness is essential to my role, in my opinion. I regularly consider my movements and demonstrations during teaching to prevent any strain, with the hope of serving as a lasting example of an active lifestyle.

### 3.2 Study 2: content validity and scale refinement

The tripartite Delphi consultation rigorously evinced robust expert consensus, with authority coefficients consistently transcending the 0.70 threshold (ranging from 0.749 to 0.764). Concomitantly, Kendall's coefficient of concordance (W) substantiated a statistically significant and ascending trajectory across successive rounds (*p* < 0.001), progressing from 0.149 in Round 1 to 0.262 in Round 3. This trajectory unequivocally underscored a progressive convergence of expert epistemologies, affirming the collective delineation of the construct.

Expert epistemological insights fundamentally reconfigured the scale's intrinsic architecture. A pivotal concern articulated during these consultations posited an initial framework that, while theoretically deductive, evinced a “technical over-specification juxtaposed with pedagogical underdevelopment.” Experts delineated that dimensions such as Motor Ability Basis were replete with granular, empirically observable sub-indicators, whereas facets concerning professional teleology and moral embodiment evinced a conceptual abstraction and a concomitant deficit in operational clarity. To redress this thematic disequilibrium and augment the scale's educational heuristic value, the framework underwent systematic reconfiguration. The original five macro-dimensions were consequently refined into a more granular and equipoised “5 Primary Dimensions × 10 Secondary Factors” matrix. Key revisions encompassed: (1) Conceptual Granularity Enhancement: the overarching construct of Embodied Moral Cultivation underwent disaggregation into discrete, empirically measurable factors, including Professional Ethics and Value Guidance, whilst Lifelong Development was meticulously operationalized into factors such as Reflection & Growth and Occupational Health. (2) Operational Definition Recalibration: the initially passive conceptualization of Blind Spot Management was proactively reconceptualized as the skill-centric factor Risk Prediction, thereby aligning the construct's operational ambit with contemporary imperatives for scholastic safety.

Subsequent to the application of stringent quantitative retention criteria (Mean >4.0, CV < 0.25) to the revised item set, the item pool underwent a successful attenuation from an initial 65 items to a refined complement of 48 items, designated for subsequent pilot assessment. This rigorous methodological filtration ensured the scale's content evinced both expert validation and intrinsic structural coherence. Whilst the Delphi rounds established a high degree of consensus regarding the inclusion of Level 1 and Level 2 dimensions, the Analytic Hierarchy Process (AHP) was subsequently employed to ascertain the logical robustness and hierarchical discrimination inherent within these constructs. Specifically, the Consistency Ratio (CR) in AHP served as a compelling proxy for structural validity, with a satisfying CR (< 0.10) corroborating experts‘ perceptions of distinct conceptual boundaries among dimensions. This methodological triangulation established that the theoretical framework was not merely broadly accepted (via Delphi) but also demonstrably logically consistent (via AHP) variance was minimal (Table 4).

**Table 4:** Analytic hierarchy process (AHP) weight distribution of PE-TEPLS dimensions.

Primary dimension (Level 1)	Weight (W1)	Secondary factor (level 2)	Local weight (W2)	Global Weight (W1 × W2)	Rank (global)
1. Teaching transformation	0.3445	Cognitive transformation	0.6	0.2067	1
		Emotional arousal	0.4	0.1378	3
2. Motor ability basis	0.2605	Core movement demonstration	0.6667	0.1737	2
		Occupational physical reserve	0.3333	0.0868	5
3. Embodied moral cultivation	0.183	Professional ethics	0.6667	0.122	4
		Value guidance & modeling	0.3333	0.061	8
4. Situational interaction	0.106	Risk prediction & first aid	0.6667	0.0707	6
		Space utilization	0.3333	0.0353	9
5. Lifelong development	0.106	Reflection & growth	0.6667	0.0707	6
		Occupational health	0.3333	0.0353	9
Consistency test				CR < 0.10	

### 3.3 Study 3: preliminary item analysis and scale refinement

A pilot investigation (*N* = 128) was conducted to evaluate the psychometric performance of the initial 48-item provisional scale. Item analysis was rigorously predicated upon Classical Test Theory (CTT) criteria, specifically the Critical Ratio (CR > 3.00) and corrected item-total correlation (*r* >0.40).

This procedure identified three problematic items—“Safe Handling” (from Occupational Physical Reserve), “Adapting to Local Conditions” (from Teaching Space), and “Tactical Visualization” (from Cognitive Transformation)—which exhibited attenuated discrimination and weak correlations with the composite score. Beyond statistical metrics, a content re-examination revealed distinct epistemological misalignments for these items.

Notably, the item “Safe Handling” (originally CB3, “I use correct posture when moving equipment”) was found to be conceptually redundant with the “Occupational Health” dimension (specifically item WH3, “Self-Protection”). Theoretical analysis suggests this item tapped into generic ergonomic safety required for manual labor, rather than the specialized embodied physical reserve unique to high-intensity pedagogical demonstration. Similarly, the item “Adapting to Local Conditions” (LY5) exhibited high collinearity with the “Environmental Adjustment” factor (e.g., adjusting for sunlight or noise), failing to provide unique variance regarding the teacher's active spatial utilization. Finally, “Tactical Visualization” (CY5), which focused on the use of static teaching boards, displayed significant cross-loading with the “Digital Demonstration” factor in the Motor Ability dimension. This blurred the structural distinction between “cognitive explanation tools” and “embodied skill demonstration.”

Consequently, the judicious removal of these three items resulted in a refined 45-item scale, sharpening the instrument's conceptual focus from generalized attributes to profession-specific embodied competencies.

### 3.4 Study 4: formal survey and psychometric validation

#### 3.4.1 Replication of factor structure (EFA)

To rigorously validate the factor architecture, the formal survey sample (*N* = 1,665) was randomly split. An Exploratory Factor Analysis (EFA) was conducted on Subsample A (*n* = 793). Addressing the theoretical expectation that embodied literacy dimensions are inherently interrelated, we employed Principal Axis Factoring (PAF) with Promax rotation. To prevent over-extraction—a common limitation of the Kaiser criterion—the number of factors was determined using Parallel Analysis (PA).

The PA results indicated that 10 factors possessed eigenvalues exceeding those from 1,000 random data permutations. The 10-factor solution explained 61.75% of the total common variance. The Pattern Matrix (Table 5) revealed a lucid structure where all 45 items loaded substantially (primary loadings >0.70) on their designated factors with minimal cross-loadings. Furthermore, the factor correlation matrix confirmed moderate inter-factor correlations (*r* range: 0.28–0.65).

**Table 5:** Pattern matrix and factor loadings for exploratory factor analysis (Subsample A, *N* = 793).

Dimensions/factors	Items	Pattern loadings^a^	Communalities (h2)^b^	Cronbach's α
1. Professional ethics	RA1	0.71	0.48	0.89
	RA2	0.81	0.61	
	RA3	0.77	0.57	
	RA4	0.77	0.58	
	RA5	0.76	0.56	
	RA6	0.74	0.51	
2. Value guidance	CF1	0.8	0.54	0.842
	CF2	0.79	0.53	
	CF3	0.81	0.56	
3. Core movement demonstration	NL1	0.72	0.51	0.916
	NL2	0.76	0.57	
	NL3	0.82	0.64	
	NL4	0.77	0.59	
	NL5	0.79	0.61	
	NL6	0.75	0.53	
	NL7	0.86	0.69	
4. Occupational physical reserve	CB1	0.74	0.5	0.858
	CB2	0.84	0.61	
	CB3	0.87	0.63	
5. Space utilization	LY1	0.81	0.57	0.852
	LY2	0.79	0.53	
	LY3	0.73	0.47	
	LY4	0.75	0.5	
6. Risk prediction	JJ1	0.79	0.56	0.88
	JJ2	0.79	0.57	
	JJ3	0.84	0.62	
	JJ4	0.81	0.57	
7. Emotional arousal	HD1	0.74	0.5	0.846
	HD2	0.77	0.51	
	HD3	0.79	0.54	
	HD4	0.75	0.5	
8. Cognitive transformation	CY1	0.72	0.49	0.854
	CY2	0.86	0.62	
	CY3	0.72	0.49	
	CY4	0.79	0.58	
9. Occupational health	WH1	0.78	0.52	0.818
	WH2	0.78	0.5	
	WH3	0.76	0.51	
10. Reflection & growth	BY1	0.74	0.56	0.92
	BY2	0.77	0.6	
	BY3	0.77	0.57	
	BY4	0.72	0.54	
	BY5	0.77	0.59	
	BY6	0.91	0.76	
	BY7	0.86	0.69	
Total variance explained				61.75%

Extraction Method: Principal Axis Factoring. Rotation Method: Promax with Kaiser Normalization.

^a^Pattern loadings represent the standardized regression coefficients indicating the unique contribution of the factor to the item. Cross-loadings below 0.30 are suppressed for clarity.

^b^Communalities (h^2^) denote the proportion of variance in each item that can be explained by the retained 10-factor solution (values > 0.40 are generally considered acceptable).

#### 3.4.2 Confirmatory factor analysis (CFA)

**Figure 2:**
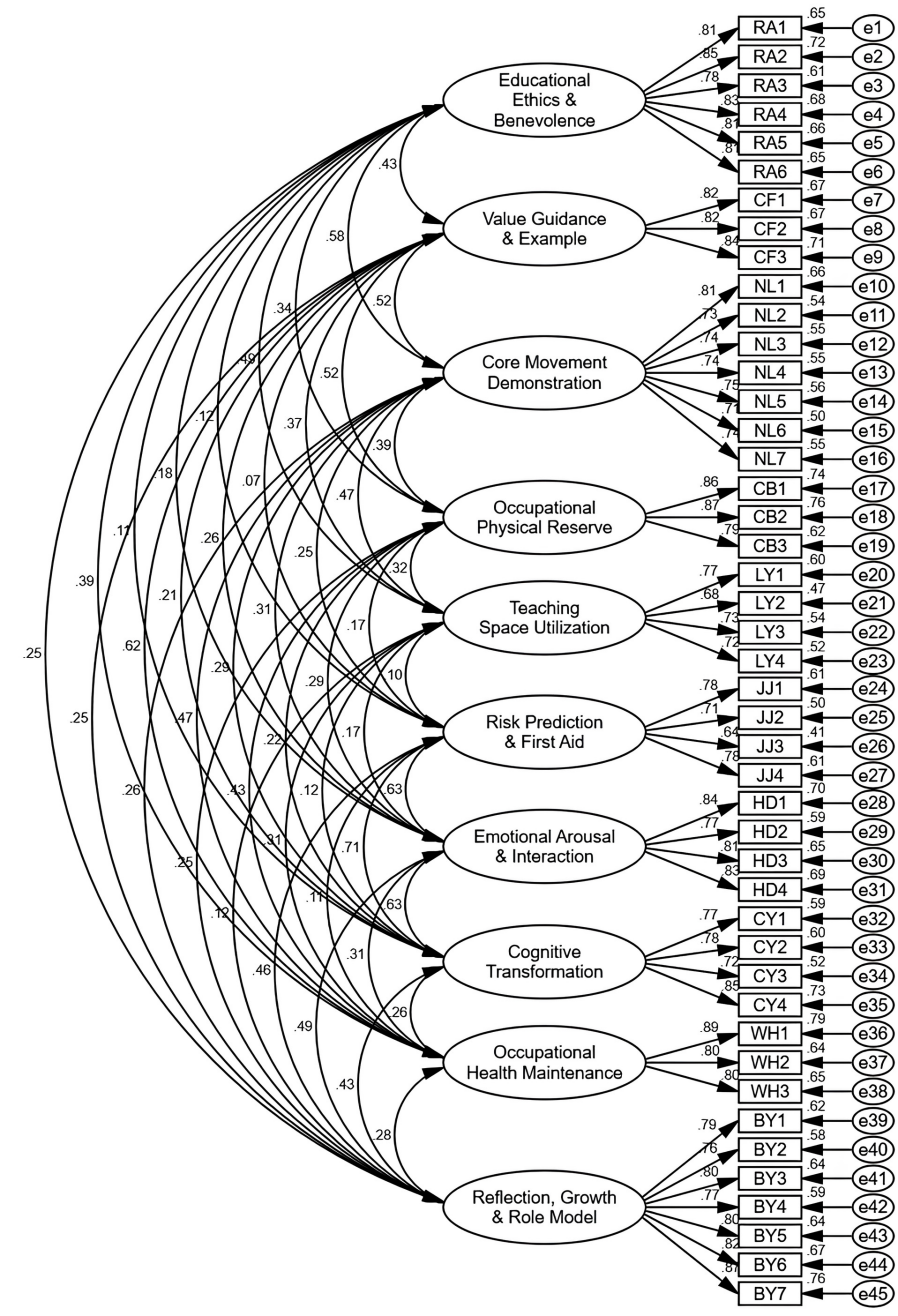
Path diagram of the confirmatory factor analysis (CFA) model.

Subsequently, a Confirmatory Factor Analysis (CFA) was executed on Subsample B (*n* = 872) utilizing AMOS 26.0. To explicitly validate the theoretical framework constructed in the Delphi phase, we specified a Second-Order Hierarchical Model. In this structure, the 45 items loaded onto 10 first-order latent factors, which in turn loaded onto 5 higher-order constructs. The results indicated that this hierarchical model achieved excellent congruence with the empirical data: χ^2^/df = 2.708 (< 3.0), CFI = 0.937, TLI = 0.930, and RMSEA = 0.044 (90% CI: 0.042–0.046). All standardized factor loadings—both from items to first-order factors and from first-order to second-order factors—were statistically significant (*p* < 0.001) and substantial (see Figure 2). This confirms that the “5-Dimension, 10-Factor” structure is a statistically valid hierarchical construct, rendering further comparison with less theoretically relevant models (e.g., unidimensional models) unnecessary.

#### 3.4.3 Psychometric properties: reliability and validity

Reliability was assessed using both Cronbach's **α** and McDonald's Omega (ω) to ensure robustness against tau-equivalence violations. As shown in Table 6, coefficients for the ten dimensions ranged from 0.818 to 0.926, indicating excellent internal consistency. Additionally, temporal stability was assessed using test-retest reliability coefficients (Pearson's *r*). Based on the subsample (*n* = 50) from the pilot phase, the results for the ten dimensions ranged from 0.773 to 0.864 (e.g., Embodied Moral Cultivation: *r* = 0.787; Occupational Health: *r* = 0.864), all exceeding the recommended threshold of 0.70. This confirms that the PE-TEPLS yields consistent measurements over time. Convergent validity was established as the Average Variance Extracted (AVE) for all dimensions exceeded the 0.50 benchmark.

**Table 6:** Summary of confirmatory factor analysis, reliability, and descriptive statistics (subsample B, *N* = 872).

Dimensions/factors	Items	Mean ±SD	Skewness/Kurtosis	Std. factor loading	Cronbach's α	CR (ρc)	AVE
1. Professional ethics	RA1	3.88 ± 0.92	−0.41/−0.47	0.807	0.921	0.938	0.717
	RA2	3.82 ± 0.96	−0.43/−0.50	0.847			
	RA3	3.80 ± 0.95	−0.47/−0.37	0.779			
	RA4	3.81 ± 0.92	−0.36/−0.45	0.826			
	RA5	3.87 ± 0.95	−0.49/−0.49	0.813			
	RA6	3.84 ± 0.93	−0.35/−0.64	0.806			
2. Value guidance	CF1	3.37 ± 0.88	−0.44/−0.50	0.819	0.867	0.919	0.79
	CF2	3.22 ± 0.90	0.02/−0.56	0.82			
	CF3	3.19 ± 0.94	−0.19/−0.63	0.845			
3. Core movement demonstration	NL1	3.87 ± 0.95	−0.67/0.08	0.812	0.898	0.92	0.622
	NL2	3.72 ± 0.94	−0.40/0.01	0.733			
	NL3	3.72 ± 0.98	−0.38/−0.41	0.741			
	NL4	3.71 ± 1.00	−0.50/−0.20	0.743			
	NL5	3.67 ± 0.98	−0.24/−0.59	0.748			
	NL6	3.67 ± 1.02	−0.59/0.14	0.708			
	NL7	3.79 ± 0.92	−0.41/−0.27	0.745			
4. Occupational physical reserve	CB1	2.75 ± 1.03	0.35/−0.51	0.858	0.877	0.924	0.803
	CB2	2.86 ± 1.14	0.05/−1.01	0.875			
	CB3	2.86 ± 0.96	0.28/−0.28	0.787			
5. Space utilization	LY1	3.66 ± 1.04	−0.76/0.24	0.774	0.818	0.877	0.641
	LY2	3.70 ± 1.04	−0.88/0.45	0.684			
	LY3	3.62 ± 1.07	−0.60/−0.19	0.734			
	LY4	3.62 ± 0.99	−0.81/0.46	0.724			
6. Risk prediction	JJ1	3.91 ± 0.78	−0.22/−0.44	0.782	0.82	0.881	0.649
	JJ2	3.59 ± 0.97	−0.12/−0.93	0.708			
	JJ3	3.58 ± 0.89	−0.17/−0.42	0.641			
	JJ4	3.77 ± 0.89	−0.21/−0.68	0.782			
7. Emotional arousal	HD1	3.70 ± 1.16	−0.54/−0.64	0.837	0.885	0.921	0.744
	HD2	3.60 ± 1.18	−0.62/−0.44	0.767			
	HD3	3.54 ± 1.08	−0.44/−0.69	0.807			
	HD4	3.69 ± 1.19	−0.60/−0.50	0.833			
8. Cognitive transformation	CY1	3.85 ± 0.95	−0.56/−0.30	0.767	0.86	0.905	0.705
	CY2	3.85 ± 1.00	−0.47/−0.79	0.776			
	CY3	3.89 ± 0.98	−0.74/0.25	0.718			
	CY4	3.78 ± 1.01	−0.35/−0.82	0.855			
9. Occupational health	WH1	3.07 ± 1.03	0.03/−0.47	0.886	0.866	0.918	0.789
	WH2	3.17 ± 1.03	−0.13/−0.77	0.798			
	WH3	3.08 ± 1.00	0.24/−0.40	0.805			
10. Reflection & growth	BY1	3.59 ± 1.05	−0.32/−0.62	0.787	0.926	0.94	0.692
	BY2	3.58 ± 1.07	−0.34/−0.59	0.765			
	BY3	3.65 ± 1.02	−0.49/−0.32	0.8			
	BY4	3.68 ± 1.06	−0.71/−0.02	0.765			
	BY5	3.57 ± 1.10	−0.42/−0.60	0.8			
	BY6	3.62 ± 1.10	−0.58/−0.31	0.818			
	BY7	3.66 ± 1.07	−0.49/−0.52	0.872			

For discriminant validity, we rigorously applied the Heterotrait–Monotrait ratio (HTMT) criterion. As presented in Table 7, all HTMT values were well below the stringent threshold of 0.85 (Range: 0.10–0.71), confirming that the latent dimensions are empirically distinct.

**Table 7:** Discriminant validity: Heterotrait–Monotrait ratio (HTMT) matrix.

Dimensions	1	2	3	4	5	6	7	8	9	10
1. Professional ethics (RA)	—									
2. Value guidance (CF)	0.433	—								
3. Core movement (NL)	0.589	0.518	—							
4. Occ. physical reserve (CB)	0.353	0.513	0.389	—						
5. Space utilization (LY)	0.497	0.373	0.486	0.332	—					
6. Risk prediction (JJ)	0.112	0.103	0.246	0.177	0.111	—				
7. Emotional arousal (HD)	0.179	0.255	0.31	0.292	0.178	0.617	—			
8. Cognitive transformation (CY)	0.107	0.217	0.291	0.226	0.122	0.706	0.636	—		
9. Occ. health (WH)	0.41	0.63	0.483	0.442	0.323	0.128	0.301	0.26	—	
10. Reflection & growth (BY)	0.25	0.247	0.261	0.251	0.125	0.47	0.495	0.442	0.283	—

#### 3.4.4 Criterion-related validity

**Figure 3:**
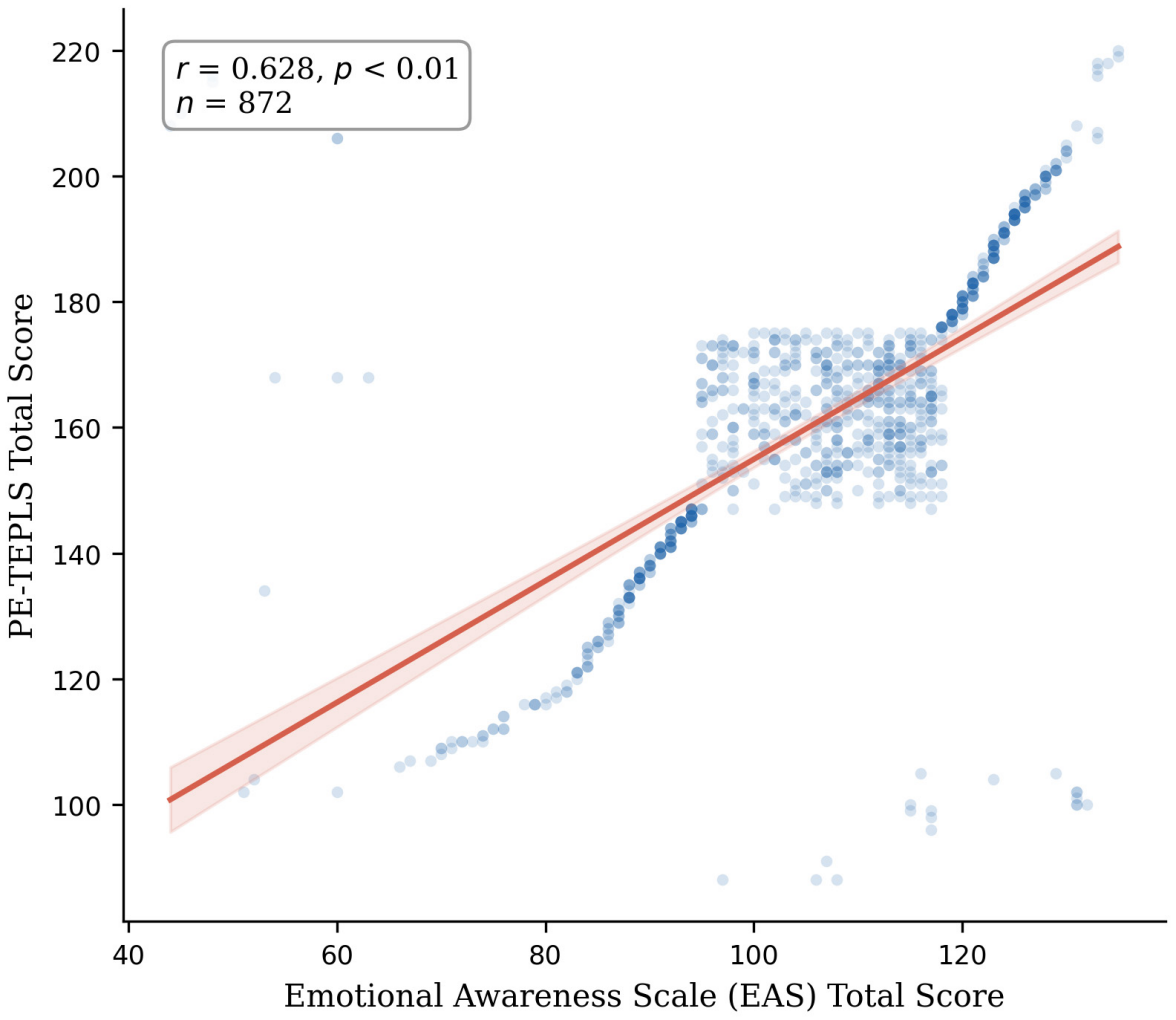
Scatter plot illustrating the correlation between PE-TEPLS and the emotional awareness scale (EAS).

The Emotional Awareness Scale (EAS) served as the criterion. Pearson correlation analysis revealed a significant, moderate-to-strong positive correlation between the PE-TEPLS and EAS total scores (*r* = 0.628, *p* < 0.01; see Figure 3). This result supports the scale's validity while demonstrating that PE-TEPLS captures a construct distinct from, yet related to, general emotional awareness.

#### 3.4.5 Measurement invariance across gender

To empirically valid the “fair ruler” claim, we conducted a Multi-Group CFA (MGCFA) across male (*n* = 563) and female (*n* = 309) teachers. We tested a hierarchy of nested models: configural invariance (baseline), metric invariance (factor loadings), structural covariance invariance, and strict invariance (measurement residuals). As detailed in Table 8, the baseline Configural Model showed excellent fit ($\frac{x^{2}}{df}$ = 1.907, IFI = 0.934), confirming that the factor structure is equivalent across genders. Subsequent constraints imposed on factor loadings (M2), structural covariances (M3), and item residuals (M4) resulted in negligible changes in fit indices. Specifically, the decrease in IFI (ΔIFI) for all model comparisons was well below the recommended cutoff of 0.010 (Cheung and Rensvold, 2002), ranging from 0.001 to 0.002. These results support strict measurement invariance, indicating that the PE-TEPLS operates equivalently for male and female teachers, thus allowing for unbiased group comparisons.

**Table 8:** Fit indices for measurement invariance models across gender (male vs. female).

Model	x^2^	df	x^2^/df	IFI	TLI	Δx^2^	Δdf	ΔIFI	Decision
M1: configural (unconstrained)	3433.08	1800	1.907	0.934	0.927	—	—	—	—
M2: metric (measurement weights)	3474.77	1835	1.894	0.933	0.928	41.69	35	0.001	Supported
M3: structural (structural covariances)	3560.82	1890	1.884	0.932	0.928	86.05	55	0.001	Supported
M4: strict (measurement residuals)	3614.38	1935	1.868	0.931	0.930	53.56	45	0.001	Supported

#### 3.4.6 Common method bias analysis

To rule out potential artifacts from self-report data, we compared the Default CFA Model with a Phantom Factor Model (ULMF). The results indicated that the inclusion of the common latent factor did not significantly improve model fit (ΔCFI = 0.006 < 0.05, ΔRMSEA = 0.001 < 0.05), and the factor loadings remained stable. These findings confirm that common method variance was minimal (Table 9).

**Table 9:** Comparison of model fit indices for CMB analysis.

Model	χ^2^/df	CFI	TLI	RMSEA	ΔCFI	ΔRMSEA
Default CFA model	2.708	0.937	0.93	0.044	—	—
ULMF (phantom) model	2.614	0.943	0.934	0.043	0.006	0.001

## 4 Discussion

### 4.1 Summary of psychometric findings and methodological advancement

The primary objective of this study was to operationalize the “pedagogical body” of K-12 PE teachers into a psychometrically valid instrument. Consistent with the view that PE teachers use their physical presence as a primary instructional tool (Silverman and Mercier, 2015), our results confirm that teacher professionalism extends beyond personal wellness to embodied enactment. Unlike verbal-centric educators, PE teachers must demonstrate motor skills and manage dynamics via non-verbal cues (Riley and Proctor, 2023). Crucially, this study advances the field methodologically by integrating the Analytic Hierarchy Process (AHP) with psychometric validation. While previous self-report measures have inherent limitations, the PE-TEPLS complements behavioral metrics by quantifying the cognitive and sensory dimensions of professional practice. By using AHP to assign weights, we move beyond simple factor identification to a hierarchically structured understanding of the “lived body” experience.

### 4.2 The ontological shift: from “athletic body” to “pedagogical body”

The structural validation of the PE-TEPLS, particularly illuminated by the Analytic Hierarchy Process (AHP) outcomes, inaugurates a seminal empirical foundation for an ontological reconfiguration in the conceptualization of physical education expertise. A pivotal finding delineates the superior salience of Teaching Transformation (Global Weight = 0.345), conspicuously transcending the contribution of Motor Ability Basis (Global Weight = 0.261). This quantitative disconfirmation robustly challenges the entrenched “dualist” paradigm (Wedgwood, 2005; Jorm, 2015), which habitually conflates a PE teacher's professional episteme with their personal athletic prowess—a reification of the “athletic body.”

Conversely, the empirical data unequivocally substantiates the conceptual paradigm of the teacher's corporeal form as a “pedagogical body”—a somatic modality principally engineered for the instructional actualization of others, rather than the idiosyncratic performativity of the self. This perspective not only aligns with but critically expands upon Shulman (1986) construct of Pedagogical Content Knowledge (PCK), extending its theoretical purview into the somatic domain. The pronounced +10.24% positive weighting shift toward pedagogical factors profoundly accentuates this recalibration: professional competence is not predicated upon superior physiological metrics, but upon the adaptive capacity for decoding and transmitting embodied knowledge.

### 4.3 The mechanism of enactment: cognitive conversion and ethical presence

Having firmly posited the epistemological primacy of the pedagogical body, a deeper critical reflection reveals the mechanisms of this enactment. First, regarding Cognitive Conversion, the deliberate retention of items quantifying the aptitude for deconstructing implicit movement knowledge (e.g., “mirror demonstration”), juxtaposed with the exclusion of generic descriptors, is significant. Analogous to findings where generalized health indices prove insufficient for differentiating specialized skill acquisition (Ou et al., 2025), we posit that rudimentary physical stamina in PE pedagogy functions merely as a “generic biological threshold.” Authentic embodied professional literacy operates not as the raw “hardware” of physical endurance, but as the sophisticated “software” (conversion aptitudes).

Second, the distinct emergence of Embodied Moral Cultivation ratifies a crucial “interactional paradigm shift.” This dimension implies that teacher ethics manifest as performative enactments rather than residing solely within abstract cognitive frameworks (Quennerstedt and Webb, 2010). In the specific cultural context of this study, this aligns with the pedagogical tradition where “body-teaching” (Shenjiao) is paramount. Within the dynamic milieu of the gymnasium, authoritative presence and nurturing solicitude are demonstrably communicated through physical comportment—such as the prophylactic utilization of one's body to shield a student—often surpassing the communicative efficacy of purely verbal instruction.

### 4.4 Demographic consistency: establishing a “fair ruler”

A persistent epistemological challenge in educational assessment resides in the pervasive potential for bias across diverse demographic cohorts (Parri and Ceciliani, 2019). Prevailing societal constructs often dichotomize male PE teachers as possessing superior “motor skills” while presuming female educators to excel in “emotional engagement” (Pautu et al., 2025). Our empirical findings directly undermine these reductionist frameworks. The rigorous Multi-Group Confirmatory Factor Analysis (MGCFA) confirmed that the PE-TEPLS achieves scalar invariance across gender (ΔCFI < 0.010, ΔRMSEA < 0.015). This established consistency bears profound implications. It unequivocally articulates that Embodied Professional Literacy constitutes a core, universal competency. Because scalar invariance was achieved, we can assert that the scale functions as an equitable “fair ruler”: a score of X represents the same level of latent ability for a female teacher as it does for a male teacher, unconfounded by gendered item functioning (Meredith, 1993; Mullen et al., 2011). This provides an objective benchmark for teacher evaluation, ensuring that assessments prioritize pedagogical competence unconfounded by personal background.

### 4.5 Strengths, limitations, and epistemological defense

This study presents notable strengths. Theoretically, it effectively operationalizes the “tacit” and “internal” dimensions of pedagogical practice. Unlike conventional scales predicating assessments upon declarative knowledge (“I feel confident...”), the PE-TEPLS targets the teacher's actualized embodied experience. Methodologically, the mixed-method design (Delphi-AHP-Psychometrics) ensures both content validity and structural hierarchy.

However, we must critically acknowledge several limitations. First, regarding the reliance on self-report data: while this approach expediently substantiates the scale, it introduces potential social desirability bias, particularly in axiologically sensitive domains like Embodied Moral Cultivation (Aquino and Reed, 2002). Nevertheless, we offer a philosophical defense: within the framework of embodied cognition, a teacher's expertise is rooted fundamentally in internal proprioception and the “first-person perspective” (Gallagher, 2005). An external observer might miss the antecedent “felt sense” (Gendlin, 1962) of a safety intervention. Therefore, while identifying it as a limitation, we also view self-report as an ontological necessity for accessing the “lived body” reality. Second, although Harman's single-factor test and the Unmeasured Latent Method Factor (ULMF) approach (Kock, 2021) attenuated immediate concerns regarding common method variance, the study remains cross-sectional. It cannot infer causal relationships between embodied literacy and student outcomes without longitudinal data. Third, the validation did not extend MGCFA to other variables such as teaching experience or urban-rural location due to sample distribution constraints (Vandenberg and Lance, 2000).

### 4.6 Future directions

Future inquiries should synergize PE-TEPLS scores with student appraisals (OECD, 2019) and objective data derived from wearable technologies or computer vision-aided video analysis. This technological triangulation would effectively bridge the gap between the teacher's subjective “felt sense” and their objective physical enactment. Furthermore, establishing the predictive linkage between PE-TEPLS scores and student learning trajectories (e.g., motor skill proficiency, affective dispositions) (Chen et al., 2017) will unequivocally reify the theoretical salience of the “pedagogical body” construct in catalyzing educational efficacy (Lambert et al., 2024).

## 5 Conclusion

In conclusion, this study successfully operationalizes the “pedagogical body” by developing and validating the Physical Education Teachers' Embodied Professional Literacy Scale (PE-TEPLS). Empirically, the study has rigorously demonstrated the scale's content validity (via Delphi-AHP), internal structural stability (via EFA and CFA), and scalar invariance across gender, establishing it as a psychometrically robust instrument for the Chinese educational context. However, it is important to distinguish these established metrics from prospective applications. While the scale's internal validity is confirmed, its predictive validity regarding student learning outcomes and its correlation with objective, non-self-report data remain critical avenues proposed for future investigation. Ultimately, the PE-TEPLS provides a necessary, theoretically aligned tool to assess the embodied professional practice of physical education teachers.

## Data Availability

The raw data supporting the conclusions of this article will be made available by the authors, without undue reservation.
