# Supplementary material for: Beyond words: developing a scale to measure the embodied professional literacy of K-12 physical education teachers
Source: Front Psychol. 2026 May 15;17:1789350. doi: 10.3389/fpsyg.2026.1789350 (PMC13218991; doi:10.3389/fpsyg.2026.1789350)
Supplement: Supplementary file 1 [file Supplementary_file_1.docx]

Appendix S1: The Physical Education Teachers’ Embodied Professional Literacy Scale (PE-TEPLS)

1. Usage Guidelines

Scoring Method: The PE-TEPLS is a self-report instrument consisting of 45 items. Each item is rated on a 5-point Likert scale:

1 = Strongly Disagree

2 = Disagree

3 = Neutral

4 = Agree

5 = Strongly Agree

Calculation: The score for each dimension is calculated as the mean of the items belonging to that dimension. A global score can be calculated by averaging all 45 items.

Interpretation: Higher scores indicate a higher level of embodied professional literacy.

Low Level: Mean score < 3.0

Moderate Level: Mean score between 3.0 and 4.0

High Level: Mean score > 4.0

2. Full Scale Items (English & Chinese Version)

| Primary Dimension | Secondary Dimension | Code | Item (English) | Item (Chinese) |
| --- | --- | --- | --- | --- |
| I. Embodied Moral Cultivation | 1. Edu-Ethics & Benevolence | RA1 | [Focus on All Students] Able to attend to all students during instruction, consciously orienting their body and gaze towards those in peripheral positions, those with physical disadvantages, or introverted students, ensuring every student feels seen and included. | [关注全体]在教学中能兼顾全体，有意识地将视线投射至边缘位置、体能弱势或性格内向的学生，确保每个学生都能感受到被关注。 |
|  |  | RA2 | [Uphold Dignity] When a student encounters an embarrassing situation (e.g., stained clothing due to menstruation, torn pants), can immediately use their own body or clothing to shield and protect the student, making every effort to preserve the student's dignity. | [维护尊严]当学生出现尴尬状况（如生理期弄脏衣物、裤子破裂）时，能第一时间用身体或衣物进行遮挡保护，全力维护学生自尊。 |
|  |  | RA3 | [Emotion Regulation] When faced with student defiance or disciplinary breaches, can strictly control physical reactions (e.g., no pushing, no pointing fingers), and employs a calm, serious posture to resolve conflicts appropriately. | [情绪管理]面对学生的顶撞或违纪行为，能严格控制肢体反应（如不推搡、不指指点点），以冷静、严肃的身体姿态稳妥化解冲突。 |
|  |  | RA4 | [Equal Communication] When conversing with a student, stops ongoing tasks and turns their body towards the student. Proactively crouches down when speaking with younger students and leans forward naturally when interacting with older students, striving to maintain eye-level contact. | [平等沟通]与学生对话时，能停下手中事务，身体转向学生。面对低年级学生会主动蹲下，与高年级学生交流时身体会自然前倾，努力保持视线平齐。 |
|  |  | RA5 | [Individualized Teaching] Able to employ appropriate body language (e.g., high-fives, thumbs-up, appropriate pats on the shoulder) tailored to students' different characteristics (including ethnic customs, personality differences, etc.) to convey acceptance, affirmation, and encouragement. | [因材施教]能根据学生的不同特点（包括民族习惯、性格差异等），运用击掌、竖大拇指、得体拍肩等适度的肢体语言，向他们传递接纳、肯定与鼓励。 |
|  |  | RA6 | [Respect Boundaries] When physical contact is necessary for correcting movements or providing protection, makes it a habit to first seek verbal or eye-contact consent from the student and strictly adheres to contact norms between teachers and students of the opposite sex. | [尊重边界]在需要进行身体接触以纠正动作或提供保护时，会养成习惯，先通过语言或眼神征得学生同意，并严格遵守异性师生的接触规范。 |
|  | 2. Value Guidance | CF1 | [Impartial Officiating] When officiating teaching competitions, uses neutral and decisive gestures and positioning to make calls; body language does not reveal any bias due to personal preference. | [执裁公正]在教学比赛执裁时，能运用中立、果断的手势和跑位进行判罚，身体语言不因个人喜好流露出任何偏袒。 |
|  |  | CF2 | [Dignified Demeanor] During solemn occasions (e.g., flag-raising ceremonies, school sports meets), maintains a standard, respectful standing posture with focused attention throughout, setting an example of patriotic etiquette for students through personal bearing. | [仪态端庄]在升旗仪式、校运会等庄重场合，能始终保持标准肃立姿态，全程神情专注，以自身身体姿态为学生树立爱国礼仪的榜样。 |
|  |  | CF3 | [Shared Adversity] Faced with harsh weather conditions (e.g., extreme cold, heat, wind/sand), persists in conducting teaching or training outdoors alongside students, avoiding seeking personal comfort shelter, thereby demonstrating requisite professional commitment. | [风雨同舟]面对严寒、酷暑、风沙等恶劣天气，能坚持与学生同在户外进行教学或训练，不独自寻找舒适处躲避，展现出应有的职业意志。 |
| II. Motor Ability Basis | 3. Core Movement Demonstration | NL1 | [Standardized Movement] Demonstrates calisthenics, school-developed exercises, and core skills with standard, fluid movements that adhere to curriculum specifications, exhibiting the inherent aesthetics and power of the actions. | [动作规范]完成广播操、校本操及各类核心技能示范时，的动作标准、舒展，符合教材规范，并体现出动作应有的美感与力度。 |
|  |  | NL2 | [Mirror Demonstration] Proficient in mirror demonstration and can flexibly use different perspectives (e.g., front, side, back) based on the cognitive characteristics of primary and secondary school students to facilitate understanding. | [镜面示范]能熟练运用镜面示范，并能根据中小学学生的认知特点，灵活采用正面、侧面或背面等不同视角进行示范，帮助学生理解。 |
|  |  | NL3 | [Bilateral Demonstration] Demonstrates normative and powerful movements with the dominant hand (e.g., right hand). Can also perform movements clearly and accurately with the non-dominant hand (e.g., left hand) as required for teaching, or skillfully uses mirror demonstration to prevent directional confusion. | [双侧展示]惯用手（如右手）动作规范、有力。在进行非惯用手（如左手）动作示范时，也能清晰、准确地完成教学所需的动作展示，或者能熟练运用“镜面示范”来帮助学生轻松理解，不混淆方向。 |
|  |  | NL4 | [Movement Deconstruction] For fast, complex sequential movements, can perform extremely slow, segmented demonstrations while effectively controlling body center of gravity to maintain stability without swaying. | [动作分解]面对快速、复杂的连贯动作，能进行极慢速的分解演示，且在过程中有效控制身体重心，保持稳定，不晃动。 |
|  |  | NL5 | [Principle Analysis] Can translate abstract principles (e.g., force sequence, biomechanics) into body metaphors easily understood by primary students (e.g., "like cracking a whip") or provide concise, intuitive mechanical analysis for secondary students. | [原理分析]能将发力顺序、身体力学等抽象原理，转化为小学生易懂的身体比喻（如“甩鞭子”），或为中学生进行简洁直观的力学分析。 |
|  |  | NL6 | [Skill Transfer] Possesses good cross-disciplinary learning ability, can quickly master core movements of emerging sports (e.g., frisbee) or traditional activities (e.g., martial arts), and effectively organize and deliver instruction. | [技能迁移]具备良好的跨项目学习能力，能快速掌握飞盘等新兴运动或武术等传统项目的核心动作，并有效组织开展教学。 |
|  |  | NL7 | [Digital Demonstration] Based on available school resources, can flexibly use electronic devices (e.g., smart wristbands, sports apps) or traditional teaching aids (e.g., tactic boards, video recordings) to visually present student movement data or complex tactical routes to facilitate learning. | [数字演示]能根据学校的现有条件，灵活使用智能手环、运动APP等电子设备，或战术板、视频录像等传统教具，将学生的运动数据或复杂的战术跑动路线直观地展示出来，帮助学生学习。 |
|  | 4. Occupational Physical Reserve | CB1 | [Adequate Endurance] Capable of consecutively delivering multiple PE classes and post-class training sessions; demonstration movements remain standard and precise even during the final stages, without showing obvious signs of fatigue. | [耐力充沛]能连续完成多节体育课及课后训练，即使在课程的最后阶段，示范动作依然标准到位，不显露明显的疲惫状态。 |
|  |  | CB2 | [Breath Control] While leading runs or demonstrating high-intensity movements, can control breathing effectively while providing clear verbal commands or explanations, ensuring actions and speech do not interfere with each other. | [呼吸讲解]在进行中长跑领跑或高强度动作示范时，能控制好呼吸，同时进行清晰的口令指挥或讲解，做到动作与语言互不干扰。 |
|  |  | CB3 | [Participatory Guidance] Based on the school level, can participate in secondary school teaching games or primary school interactive play, providing real-time tactical guidance or enlivening the classroom atmosphere through personal involvement. | **[参与指导]**能根据学段特点，参与中学的教学比赛或小学的游戏互动，并通过亲身参与，进行实战战术指导或活跃课堂氛围。 |
| III. Situational Interaction | 5. Teaching Space | LY1 | [Environmental Regulation] Proactively adjusts team orientation and personal positioning to have students stand with their backs to strong light or wind/sand, and uses positional advantages to minimize interference from environmental noise (e.g., traffic) on the class. | [环境调控]能主动调整队伍朝向和自身站位，让学生背对强光或风沙，并利用位置优势减小环境噪音（如马路声）对课堂的干扰。 |
|  |  | LY2 | [Sightline Coverage] During group instruction, ensures an unobstructed view for every student by adjusting formation (e.g., having front rows crouch) or standing on elevated ground, so all can see the demonstration clearly. | [视线覆盖]在集合讲解时，能通过调整队形（如让前排蹲下）或站到高处，确保每位学生的视线都不受阻挡，都能看清示范。 |
|  |  | LY3 | [Zonal Isolation] Can use body positioning or markers (e.g., cones) to establish clear "safety zones," effectively isolating adjacent classes to prevent incidents like stray ball injuries. | [区域隔离]能利用身体站位或标志物（如锥桶）设置清晰的“安全警戒区”，有效隔离相邻班级，预防球类等误伤事件。 |
|  |  | LY4 | [Global Observation] During instruction, habitually positions themselves at the periphery of the area (back against a wall or corner), avoiding the center where blind spots occur behind, to facilitate comprehensive observation of all students. | [全局观察]在教学过程中，习惯处于场地边缘（背靠围墙或角落），避免身处场地中心造成背后盲区，以便全面观察所有学生。 |
|  | 6. Risk Prediction | JJ1 | [Keen Observation] Possesses sharp observational skills to quickly identify potential safety hazards on students (e.g., loose shoelaces, sharp accessories) and immediately pause activities for correction. | [明察秋毫]具备敏锐的观察力，能迅速发现学生身上如鞋带松散、佩戴尖锐饰品等安全隐患，并立即暂停活动予以纠正。 |
|  |  | JJ2 | [Attentive Monitoring] Can keenly identify abnormal signs of students with sensitive constitutions or excessive fatigue through observing student complexion, lip color, breathing rate, etc., and take timely intervention measures. | [体察入微]能通过观察学生的面色、唇色、呼吸频率等，敏锐识别出特异体质或过度疲劳学生的异常表现，并及时采取干预措施。 |
|  |  | JJ3 | [Conflict Intervention] During confrontational drills, when noticing signs of potential conflict between students, can quickly intervene by positioning their body between parties, using posture for effective isolation and mediation. | [冲突干预]在对抗性练习中，当发现学生有冲突苗头时，能迅速切入双方之间，利用身体姿态进行有效的隔离与劝阻。 |
|  |  | JJ4 | [Proficient First Aid] Masters emergency response skills for common sports injuries—including standardized, confident CPR, AED use, and handling nosebleeds and sprains—performing them proficiently and decisively. | [急救娴熟]熟练掌握心肺复苏（CPR）、AED使用，以及流鼻血、扭伤等常见运动损伤的应急处理技能，且操作规范、果断。 |
| IV. Teaching Transformation | 7. Emotional Arousal | HD1 | [Professional Demeanor] Stands upright with an energetic and full spirit, eyes firm and bright, exuding the vitality expected of a PE teacher, avoiding slack postures like arms akimbo or leaning. | [教态端正]站立时身体挺拔、精神饱满，目光坚定有神，展现出体育教师应有的朝气，无叉腰、斜靠等松懈姿态。 |
|  |  | HD2 | [Non-Verbal Management] Skillfully uses non-verbal cues—eye contact, facial expressions, gesture commands—to effectively maintain classroom discipline and focus student attention without raising their voice in criticism. | [无声管理]能熟练运用眼神交流、表情提示、手势指令等非语言方式，在不高声批评的情况下，有效维持课堂纪律并集中学生注意力。 |
|  |  | HD3 | [Whistle Application] Employs professional whistle sounds with variations in length and intensity to replace repetitive verbal commands, enabling quick and efficient team management in large classes or noisy environments. | [口哨运用]能运用长短变化、强弱有序的专业哨音，替代重复口令，在大班教学或嘈杂场地中实现快速、高效的队伍调度。 |
|  |  | HD4 | [Strong Engagement] Uses energetic body movements (e.g., appropriately exaggerated demonstrations, firm high-fives) and clear, loud voices to stimulate student participation, balancing fun for primary years with motivational guidance for secondary years. | [感染力强]能通过富有活力的肢体动作（如适当夸张的演示、有力击掌）和洪亮清晰的声音，调动学生的参与热情，兼顾小学阶段的趣味性与中学阶段的激情引导。 |
|  | 8. Cognitive Transformation | CY1 | [Smooth Workflow] During transitions between teaching phases (e.g., instruction, practice, competition), uses clear body movement paths and explicit gestures to achieve natural, seamless classroom organization. | **[流程顺畅]**在“学、练、赛”等教学环节转换时，能通过清晰的身体移动路线和明确手势，实现课堂组织自然流畅、衔接无缝。 |
|  |  | CY2 | [Differentiated Guidance] Can promptly demonstrate alternative movements with appropriate difficulty levels for students with weaker physical abilities or special needs, reflecting awareness of individualized instruction. | [分层指导]能针对体能较弱或有特殊需要的学生，及时展示难度适宜的替代性动作，体现因材施教的教学意识。 |
|  |  | CY3 | [Effective Error Correction] When imitating incorrect movements for correction, focuses on technical comparison rather than personal mimicry, avoiding mocking imitation targeted at specific students to protect their self-esteem and confidence. | [纠错有方]在模仿错误动作进行纠正时，注重技术对比而非个人模仿，避免针对具体学生进行滑稽演示，保护学生自尊与信心。 |
|  |  | CY4 | [Synchronized Explanation] Coordinates verbal explanations with movement demonstrations; using "demonstration while explaining" to help students easily grasp key points, avoiding disconnection between explanation and action. | [讲做同步]讲解与动作示范协调一致，通过“边讲边做”帮助学生轻松理解动作要领，避免先讲后做、讲做脱节。 |
| V. Lifelong Development | 9. Occupational Health | WH1 | [Scientific Voice Use] Proficient in using diaphragmatic breathing for vocalization, adept at using whistles and gestures as substitutes for shouting, effectively preventing throat discomfort. | [科学用嗓]能熟练运用腹式呼吸法发声，善于利用哨音、手势等替代高声喊叫，有效预防咽喉不适。 |
|  |  | WH2 | [Proper Protection] When handling equipment or providing spotting, follows correct posture for exertion (e.g., bending knees to lift heavy objects), paying attention to protecting joints like the waist and knees to prevent occupational strain. | [防护有方]在搬运器材或进行保护时，能遵循正确用力姿势（如屈膝搬重物），注意保护腰膝等关节，预防职业劳损。 |
|  |  | WH3 | [Emotional Support] When a student is emotionally agitated or overly tense, can provide a sense of security and help stabilize their emotions through a calm posture and appropriate close proximity. | [情绪支持]当学生情绪激动或过度紧张时，能通过平静的身体姿态和适当的近距离陪伴，给予安全感，帮助学生稳定情绪。 |
|  | 10. Reflection & Growth | BY1 | [Posture Observation] Can promptly identify abnormalities in student body posture (e.g., uneven shoulders, tendency to slouch) and offer reminders for correct posture and daily improvement suggestions. | [体态观察]能及时发现学生身体姿态异常（如高低肩、驼背倾向），并给予正确姿势的提醒和日常改善建议。 |
|  |  | BY2 | [Discernment of Claims] Can use experience to assess the plausibility of student claims of "physical discomfort," balancing the protection of student dignity with not overlooking potential health risks. | [明辨真伪]能结合经验判断学生“身体不适”陈述的合理性，既保护学生自尊，也不忽视潜在健康风险。 |
|  |  | BY3 | [Exemplary Appearance] Dresses professionally and neatly for class, with a tidy hairstyle; avoids teaching in leather shoes or casual wear, setting a healthy example through personal conduct. | [仪表示范]上课时衣着专业整洁，发型利落，不穿皮鞋或便装上课，以身作则树立健康形象。 |
|  |  | BY4 | [Reflective Improvement] Can identify personal habitual minor movements or ineffective behaviors by reviewing class recordings or seeking colleague feedback, and consciously works on improvement. | [反思精进]能通过回看课堂录像或听取同事反馈，察觉自身习惯性小动作或无效行为，并有意识改进。 |
|  |  | BY5 | [Lifelong Exercise] Maintains regular exercise habits, sustaining good physique and motor skills, inspiring students to love sports through a positive, healthy image. | [终身锻炼]保持规律运动习惯，维持良好体态与运动能力，以积极健康的形象感染学生热爱体育。 |
|  |  | BY6 | [Interdisciplinary Integration] When explaining movements, can borrow knowledge from other disciplines (e.g., using "leverage" for force, "rhythm" for cadence), "translating" these principles into body movements to help students understand. | [融会贯通]在讲解动作时，我能借用其他学科的知识（如用“杠杆”讲发力，用“节奏”讲韵律），把这些原理“翻译”成身体动作，帮助学生更深入地理解要领。 |
|  |  | BY7 | [Health Guidance] Possesses public health awareness, can demonstrate protective actions standardly, and rationally organizes low-contact physical activities that meet health requirements. | [卫生引导]具备公共卫生意识，能规范演示防护动作，合理组织低接触、符合健康要求的体育活动。 |
